# Renal function is associated with one-month and one-year mortality in patients with intracerebral hemorrhage

**DOI:** 10.1371/journal.pone.0269096

**Published:** 2023-01-26

**Authors:** I-Kuan Wang, Tzung-Hai Yen, Chon-Haw Tsai, Yu Sun, Wei-Lun Chang, Po-Lin Chen, Ta-Chang Lai, Po-Yen Yeh, Cheng-Yu Wei, Cheng-Li Lin, Kai-Cheng Hsu, Chi-Yuan Li, Fung-Chang Sung, Chung Y. Hsu, Taiwan Stroke Registry Investigators

**Affiliations:** 1 Department of Medicine, College of Medicine, China Medical University, Taichung, Taiwan; 2 Division of Nephrology, China Medical University Hospital, Taichung, Taiwan; 3 Division of Nephrology, Chang Gung Memorial Hospital, Taipei, Taiwan; 4 Chang Gung University College of Medicine, Taoyuan, Taiwan; 5 Department of Neurology, China Medical University Hospital, Taichung, Taiwan; 6 Neurology, En Chu Kong Hospital, New Taipei City, Taiwan; 7 Department of Neurology, College of Medicine, National Cheng Kung University, Tainan, Taiwan; 8 Department of Neurology, Show Chwan Memorial Hospital, Changhua County, Taiwan; 9 Neurological Institute, Taichung Veterans General Hospital, Taichung, Taiwan; 10 Department of Neurology, School of Medicine, National Yang-Ming University, Taipei, Taiwan; 11 Division of Neurology Department of Internal Medicine, Cheng Hsin General Hospital, Taipei, Taiwan; 12 Department of Neurology, St. Martin De Porres Hospital, Chiayi, Taiwan; 13 Department of Neurology, Chang Bing Show Chwan Memorial Hospital, Changhua, Taiwan; 14 Management Office for Health Data, China Medical University Hospital, Taichung, Taiwan; 15 Department of Anesthesiology, China Medical University Hospital, Taichung, Taiwan; 16 Graduate Institute of Biomedical Science, China Medical University College of Medicine, Taichung, Taiwan; 17 Department of Health Services Administration, China Medical University College of Public Health, Taichung, Taiwan; 18 Department of Food Nutrition and Health Biotechnology, Asia University, Taichung, Taiwan; Osaka University Graduate School of Medicine, JAPAN

## Abstract

**Objective:**

This study evaluated short-term (1-month) and long-term (1-year) mortality risks associated with the glomerular filtration rate (eGFR) on admission for patients with intracerebral hemorrhage.

**Methods:**

From the Taiwan Stroke Registry data from April 2006 to December 2016, we identified and stratified patients with intracerebral hemorrhage into five subgroups by the eGFR level on admission: ≥90, 60–89, 30–59, 15–29, and <15 mL/min/1.73 m^2^ or on dialysis. Risks for 1-month and 1-year mortality after intracerebral hemorrhage were compared by the eGFR levels.

**Results:**

Both the 1-month and 1-year mortality rates progressively increased with the decrease in eGFR levels. The 1-month mortality rate in patients with eGFR < 15 mL/min/1.73 m^2^ or on dialysis was approximately 5.5-fold greater than that in patients with eGFR ≥ 90 mL/min/1.73 m^2^ (8.31 versus 1.50 per 1000 person-days), with an adjusted hazard ratio (HR) of 4.59 [95% confidence interval (CI) = 2.71–7.78]. Similarly, the 1-year mortality in patients with eGFR < 15 mL/min/1.73 m^2^ or on dialysis was 7.5 times that in patients with eGFR ≥ 90 mL/min/1.73 m^2^ (2.34 versus 0.31 per 1000 person-days), with an adjusted HR of 4.54 (95% CI 2.95–6.98).

**Conclusion:**

Impairment of renal function is an independent risk factor for mortality in patients with intracerebral hemorrhage in a gradual way. The eGFR level is a prognostic indicator for patients with intracerebral hemorrhage.

## Introduction

Stroke is one of the major causes of death and disability worldwide, and it has become the fourth leading cause of death in the USA [1]. Stroke has tremendous effects on the quality of life, productivity, and health care costs. Thus, it is important to examine the prognostic factors for patients with stroke. Identifying patients at a higher risk of death after stroke could benefit healthcare providers in patient care.

Renal dysfunction is one of the risk factors for cardiovascular complications, including stroke and death [2–5]. On the contrary, renal function impairment is highly prevalent in patients with stroke. Up to 50% of stroke patients have preexisting renal dysfunction [6, 7]. Previous studies have revealed that renal dysfunction predicts risks of both mortality and cardiovascular complications in patients with stroke [7–9]. A graded relationship is found between renal dysfunction and cardiovascular outcomes, including deaths in these patients. In our previous studies, we found that the estimated glomerular filtration rate (eGFR) is associated with risks of both 1-month and 1-year mortality and recurrence in patients with acute ischemic stroke [10, 11]. Intracerebral hemorrhage is a more devastating type of stroke than ischemic stroke, carrying a higher risk of subsequent morbidity and mortality [12, 13]. Renal dysfunction is a poor prognostic factor in patients with intracerebral hemorrhage [14–18]. However, other studies have failed to demonstrate renal dysfunction as a significant predictor of mortality in patients with intracerebral hemorrhage [8, 19]. Because of previous inconsistent results, this study investigated whether the risks of 1-month and 1-year mortality are associated with eGFR levels in patients with intracerebral hemorrhage using the Taiwan Stroke Registry (TSR) database.

## Methods

### Data source

TSR is a multicenter stroke registry system, launched in 2006, with 38 hospitals (16 medical centers, 20 regional hospitals, and 2 local hospitals) participating in this program [20]. Trained personnel at participating hospitals performed registry tasks and collected follow-up data. The registration data contained demographic profiles, National Institute of Health Stroke Scale (NIHSS) score, hospitalization records, discharge information, and follow-up data. Telephone calls were performed at 1, 3, 6, and 12 months after stroke to collect follow-up information, including deaths. The informed consent forms were obtained from all patients for being included in the registry program.

This study was performed in compliance with guidelines of the Declaration of Helsinki and approved by the Research Ethics Committee of China Medical University Hospital [CMUH102-REC1-086 (CR3)].

### Study population

**Fig 1:**
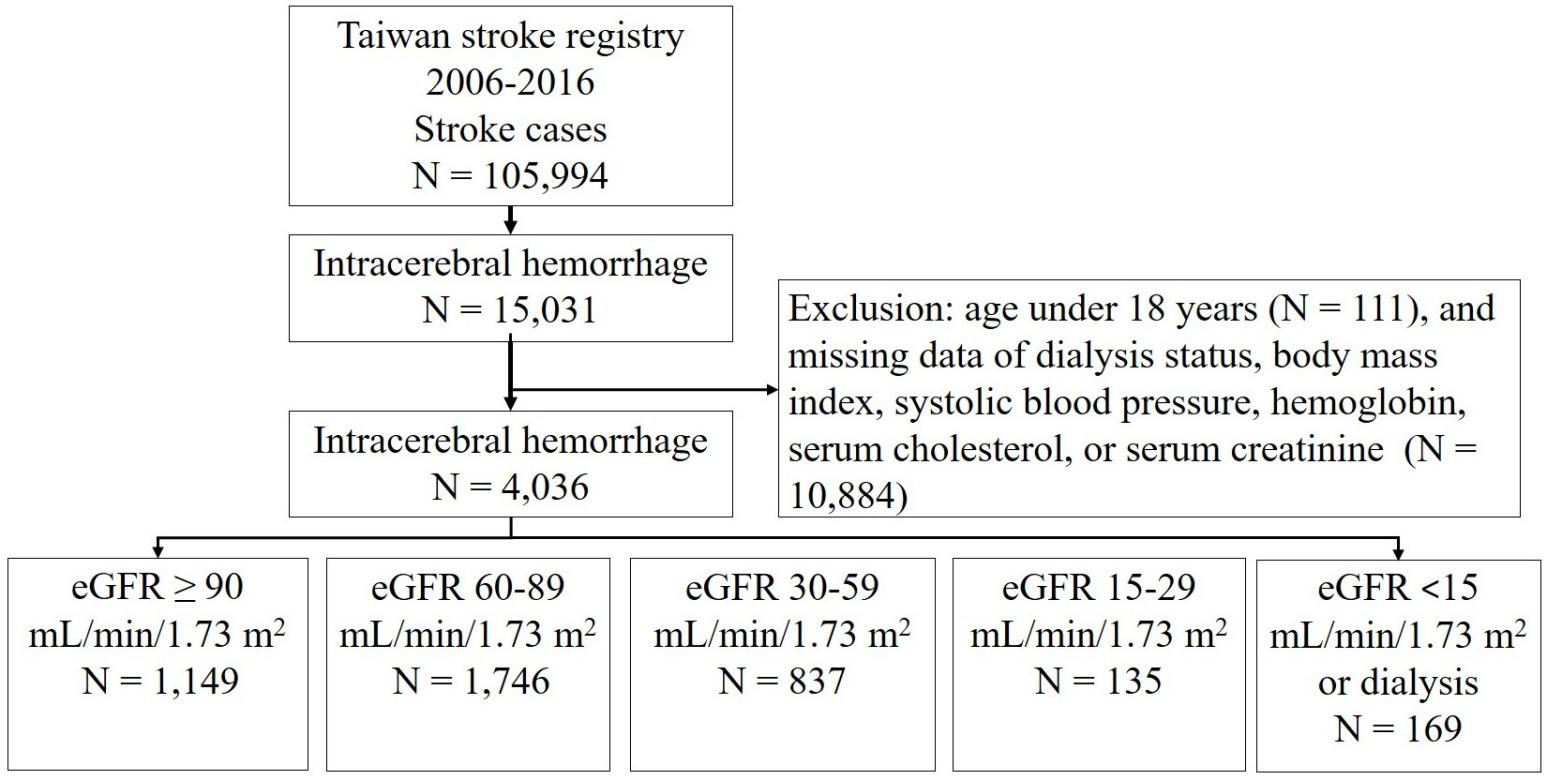
Flow chart for identifying patients with intracerebral hemorrhage by eGFR levels on admission.

Among a total of 105,994 patients with stroke registered from 2006 to 2016 in the TSR, 15,031 has intracerebral hemorrhage (Fig 1). Patients with intracerebral hemorrhage caused by trauma or brain tumors were not registered in this database. We excluded patients aged < 18 years, and patients without information on dialysis status, body mass index (BMI), systolic blood pressure levels, hemoglobin (Hb) levels, serum cholesterol levels, or serum creatinine levels. Patients who died after withdrawal of life-sustaining measures were also included in this study. A total of 4,036 patients with hemorrhagic stroke were included in this study and divided into five subgroups by the level of the estimated glomerular filtration rate (eGFR): ≥90, 60–89, 30–59, 15–29, and <15 mL/min/1.73 m^2^ or on dialysis. Patients with eGFR ≥ 90 mL/min/1.73 m^2^ were selected as the control group. Patients on dialysis were classified to be in stage 5D of chronic kidney disease [21]. Thus, patients with eGFR < 15 mL/min/1.73 m^2^ or on dialysis were combined into one group. The eGFR was calculated by the Chronic Kidney Disease Epidemiology Collaboration (CKD-EPI) equation for each patient not on dialysis [22]. The CKD-EPI equation, expressed as a single equation, is calculated as follows: GFR = 141 × min (Scr/κ, 1)^α^ × max (Scr/κ, 1)^−1.209^ × 0.993^Age^ × 1.018 [if female] × 1.159 [if black], where Scr is the serum creatinine level, κ is 0.7 for women and 0.9 for men, α is −0.329 for women and −0.411 for men, min indicates the minimum of Scr/κ or 1, and max indicates the maximum of Scr/κ or 1. The etiologies of intracerebral hemorrhage were classified into hypertensive vasculopathy and non-hypertensive causes such as amyloid angiopathy, ruptured vascular malformation, and aneurysm. The 1-month and 1-year mortality rates after intracerebral hemorrhage were evaluated among the five groups based on eGFR levels.

### Statistical analysis

Distributions of baseline characteristics were compared among the five eGFR groups, by sex, age, BMI, smoking, comorbidity, clinical characteristics, and medication use before admission. Continuous variables were expressed as median, 25th percentile, and 75th percentile and examined using the Kruskal–Wallis test for continuous variables not normally distributed. Categorical variables were expressed as frequencies and examined using the Chi-square test.

The cumulative mortality rates of the five eGFR subgroups were plotted by the Kaplan–Meier method and tested using the log-rank test. The 1-month and 1-year mortality rates (per 1000 person-days) after intracerebral hemorrhagic were calculated by the eGFR levels. We further used the Cox proportional hazards regression analysis to estimate the hazard ratio (HR) of mortality and 95% confidence interval (CI) associated with eGFR levels on admission for patients with intracerebral hemorrhage, using the subgroup with eGFR ≥ 90 mL/min/1.73 m^2^ as reference. Adjusted HRs were further calculated using multivariate Cox regression models after controlling for all covariates. We also used the receiver operating characteristic (ROC) curve to assess the predictive performance of eGFR levels with regard to 1-month and 1-year mortality after intracerebral hemorrhage. All statistical analyses were performed using SAS statistical software, version 9.4 (SAS Institute Inc., Cary, NC). Statistical significance was defined using two-tailed tests (P < 0.05).

## Results

Among 4,036 patients with intracerebral hemorrhage, 2887 (71.5%) had eGFR < 90 mL/min/1.73 m^2^, and 169 (4.2%) had eGFR < 15 mL/min/1.73 m^2^ or on dialysis (Fig 1). A total of 103 patients underwent dialysis.

As shown in Table 1, more than two-thirds (n = 2677) of the patients with intracerebral hemorrhage were men. The age of the study population increased as the eGFR levels decreased, with a median age of 61.4 years. In general, the prevalence rates of comorbidities, NIHSS scores, systolic blood pressure, and medication use before admission tended to be higher in groups with lower eGFR, whereas Hb levels were lower in groups with lower eGFR. Hypertension was the most prevalent comorbidity (86.6%) in all patients, followed by diabetes mellitus, previous stroke, ischemic heart disease, atrial fibrillation, and congestive heart failure (1.21%).

**Table 1:** Baseline characteristics of patients with intracerebral hemorrhage by eGFR levels on admission.

	eGFR mL/min/1.73 m^2^												
Variable	Total N = 4036		≧90 N = 1149 (28.5%)		60–89 N = 1746 (43.3%)		30–59 N = 837 (20.7%)		15–29 N = 135 (3.34%)		<15 or dialysis N = 169(4.19%)		p-value
Men, N (%)	2677	(66.3)	749	(65.2)	1174	(67.2)	573	(68.5)	82	(60.7)	99	(58.6)	0.05*
Age, year, median (Q1, Q3)	61.4	(51.7, 72.9)	54.2	(46.1, 61.4)	63.3	(53.2, 74.1)	71.0	(58.7, 80.2)	70.0	(58.5, 79.6)	60.5	(52.6, 69.2)	<0.0001^#^
BMI, kg/m^2^, median (Q1, Q3)	24.4	(21.8, 27.5)	24.7	(22.1, 27.6)	24.4	(22.0, 24.7)	24.2	(21.5, 27.1)	24.0	(20.6, 26.6)	22.8	(20.1, 25.8)	<0.0001^#^
Smoking													<0.0001*
Current	1061	(26.3)	384	(33.4)	450	(25.8)	164	(19.6)	29	(21.5)	34	(20.1)	
Past	408	(10.1)	92	(8.01)	185	(10.6)	105	(12.5)	13	(9.63)	13	(7.69)	
Etiology													0.31
Hypertensive	3351	(83.0)	936	(81.5)	1458	(83.5)	697	(83.3)	112	(83.0)	148	(87.6)	
Non-hypertensive	685	(17.0)	213	(18.5)	288	(16.5)	140	(16.7)	23	(17.0)	21	(12.4)	
Comorbidity, N (%)													
Hypertension	3496	(86.6)	943	(82.1)	1517	(86.9)	757	(90.4)	120	(88.9)	159	(94.1)	<0.0001
AF	102	(2.53)	18	(1.57)	36	(2.06)	32	(3.82)	13	(9.63)	3	(1.78)	<0.0001*
Previous stroke history	739	(18.3)	140	(12.2)	333	(19.1)	197	(23.5)	38	(28.2)	31	(18.3)	<0.0001*
Ischemic heart disease	298	(7.38)	47	(4.09)	108	(6.19)	99	(11.8)	18	(13.3)	26	(15.4)	<0.0001*
Congestive heart failure	49	(1.21)	7	(0.61)	12	(0.69)	15	(1.79)	9	(6.67)	6	(3.55)	<0.0001*
Diabetes mellitus	1048	(26.0)	259	(22.5)	384	(22.0)	254	(30.4)	66	(48.9)	85	(50.3)	<0.0001*
Systolic blood pressure, mmHg	177.0	(153.0, 203.0)	170.0	(148.0, 196.0)	179.0	(155.0, 202.0)	181.0	(157.0, 211.0)	184.0	(153.0, 210.0)	187.0	(160.0, 215.0)	<0.0001^#^
Laboratory data, median (Q1, Q3)													
Cholesterol, mg/dl	150.0	(104.0, 192.0)	156.0	(112.0, 197.0)	148.0	(100.0, 192.0)	146.0	(100.0, 187.0)	145.0	(110.0, 205.0)	150.0	(108.0, 191.0)	0.37^#^
Hb, g/dL	14.2	(12.7, 15.4)	14.6	(13.3, 15.6)	14.4	(13.2, 15.5)	13.7	(12.3, 15.2)	11.8	(10.4, 13.3)	10.6	(9.30, 11.6)	<0.0001^#^
NIHSS score on admission, median (Q1, Q3)	8.00	(3.00, 17.0)	8.00	(3.00, 16.0)	8.00	(3.00, 15.0)	9.00	(3.00, 21.0)	12.0	(4.00, 29.0)	11.0	(4.00, 25.0)	<0.0001^#^
Medicine use before admission, N (%)													
Antiplatelet drugs	367	(9.09)	63	(5.48)	162	(9.28)	103	(12.3)	20	(14.8)	19	(11.2)	<0.0001*
Warfarin	81	(2.01)	11	(0.96)	31	(1.78)	27	(3.23)	9	(6.67)	3	(1.78)	0.004*
Lipid lowering drugs	204	(5.05)	42	(3.66)	78	(4.47)	52	(6.21)	18	(13.3)	14	(8.28)	<0.0001*

*Chi-square test, and

^#^Kruskal–Wallis test

eGFR, estimated glomerular filtration rate; Q1, 25th percentile; Q3, 75th percentile; BMI, body mass index; AF, atrial fibrillation; Hb, hemoglobin; NIHSS, National Institutes of Stroke Scale

**Fig 2:**
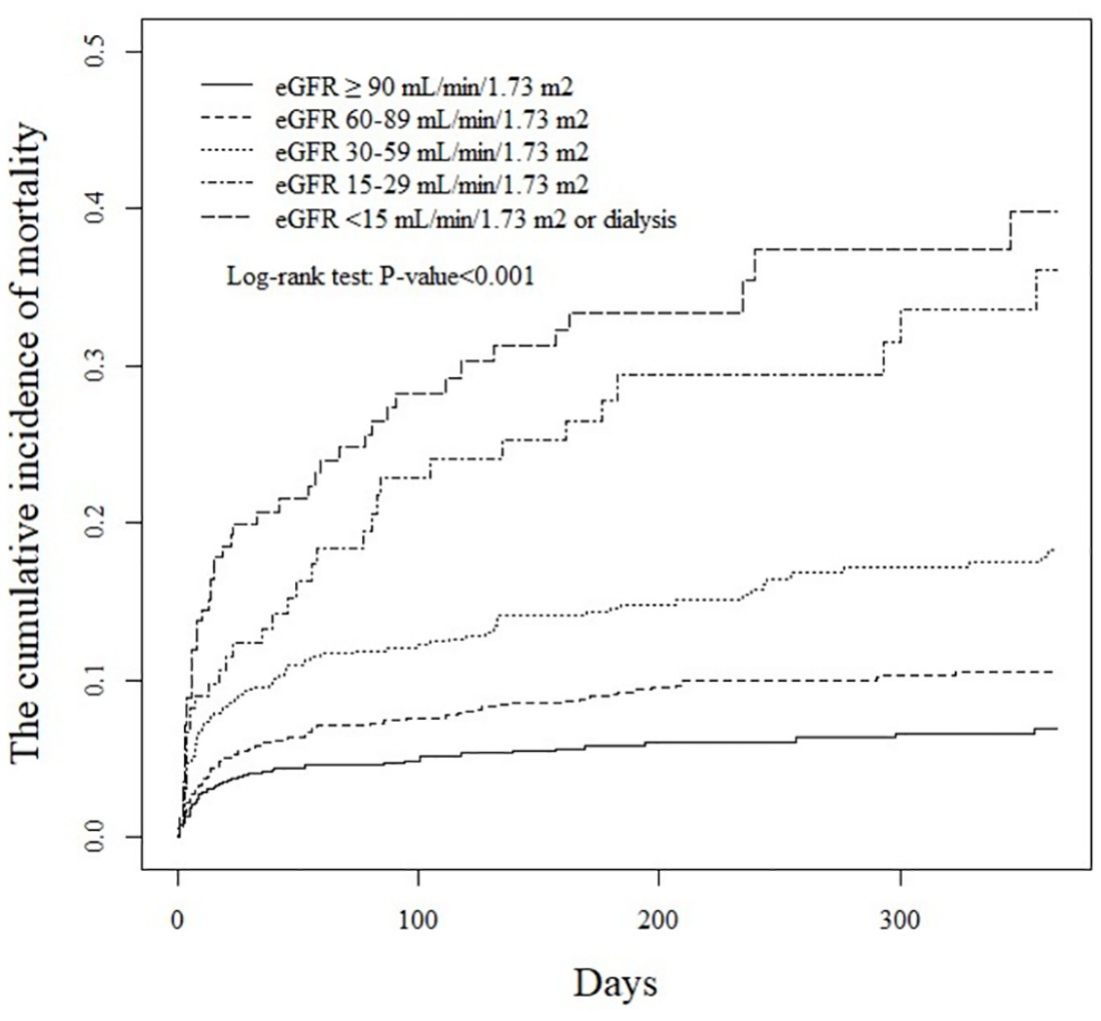
The cumulative incidence of mortalities after intracerebral hemorrhagic by eGFR levels on admission.

The cumulative 1-year overall mortality after intracerebral hemorrhage increased as the eGFR level declined (*P* < 0.001). The mortality rate in the group with eGFR < 15 mL/min/1.73 m^2^ was 38% greater than that in the group with eGFR ≥ 90 mL/min/1.73 m^2^ (Fig 2).

Both 1-month mortality (N = 258) and 1-year mortality (N = 394) rates in patients with intracerebral hemorrhage were negatively associated with their eGFR levels on admission (Tables 2 and 3). The 1-month mortality rate increased from 1.5 per 1000 person-days in patients with eGFR ≥ 90 mL/min/1.73 m^2^ to 8.31 per 1000 person-days in patients with eGFR < 15 mL/min/1.73 m^2^ or on dialysis. The adjusted HR was 4.59 (95% CI = 2.71, 7.78) for those with eGFR < 15 mL/min/1.73 m^2^ compared with those with eGFR ≥ 90 mL/min/1.73 m^2^. The corresponding one-year mortality rates were 0.31 and 2.34 per 1000 person-days, respectively, with an adjusted HR of 4.54 (95% CI = 2.59, 6.98) for patients with eGFR < 15 mL/min/1.73 m^2^ or on dialysis. The in-hospital mortality rates for patients with intracerebral hemorrhage were also negatively associated with their eGFR levels on admission with 3.9-fold higher in the group with eGFR < 15 mL/min/1.73 m^2^ than that in the group with ≥ 90 mL/min/1.73 m^2^ (Table 4). In addition, the mortality rate associated with the withdrawal of life support in patients with eGFR < 15 mL/min/1.73 m^2^ was 11.7-fold greater than those with eGFR ≥ 90 mL/min/1.73 m^2^. Table 5 presents that the 1-year mortality after intracerebral hemorrhage was associated with female sex, age, non-hypertensive cause, atrial fibrillation, systolic blood pressure, and NIHSS score on admission. The use of antiplatelet drugs before admission was associated with a lower risk of 1-year mortality after intracerebral hemorrhage.

**Table 2:** Incidence and hazard ratios of mortality at 1 month after intracerebral hemorrhage by eGFR levels on admission.

	Mortality N = 258	Person-days	Rate^‡^	Crude HR (95% CI)	Adjusted HR (95% CI)^a^
eGFR mL/min/1.73 m^2^	n				
≥ 90	43	28667	1.50	1.00	1.00
60–89	93	43836	2.12	1.42(0.89, 2.04)	1.54(1.05, 2.27)*
30–59	74	20265	3.65	2.42(1.66, 3.53)**	2.24(1.48, 3.38)***
15–29	16	3332	4.80	3.22(1.81, 5.71)**	2.22(1.20, 4.12)*
<15 or dialysis	32	3849	8.31	5.40(3.42, 8.54)**	4.59(2.71, 7.78)***
P for trend			<0.001	<0.001	<0.001

^‡^per 1000 person-days

^a^Adjusted for age, sex, body mass index, smoking, etiology of intracerebral hemorrhage, hypertension, atrial fibrillation, previous stroke history, ischemic heart disease, congestive heart failure, diabetes mellitus, systolic blood pressure, hemoglobin, cholesterol, NIHSS score on admission, and medication use before admission

*p < 0.05,

**p < 0.01,

***p < 0.001

**Table 3:** Incidence and hazard ratios of mortality at 1 year after intracerebral hemorrhage by eGFR levels on admission.

	Mortality N = 394	Person-days	Rate^‡^	Crude HR (95% CI)	Adjusted HR (95% CI)^a^
All-cause mortality					
eGFR mL/min/1.73 m^2^					
≥ 90	59	187392	0.31	1.00	1.00
60–89	139	299308	0.46	1.52(1.12, 2.06)*	1.49(1.08, 2.04)*
30–59	112	134013	0.84	2.66(1.94, 3.65)**	2.06(1.47, 2.89)***
15–29	34	19728	1.72	5.12(3.36, 7.81)**	3.02(1.91, 4.77)***
<15 or dialysis	50	21375	2.34	6.48(4.44, 9.44)**	4.54(2.95, 6.98)***
P for trend			<0.001	<0.001	<0.001

^‡^per 1000 person-days

^a^Adjusted for age, sex, body mass index, smoking, etiology of intracerebral hemorrhage, hypertension, atrial fibrillation, previous stroke history, ischemic heart disease, congestive heart failure, diabetes mellitus, systolic blood pressure, hemoglobin, cholesterol, NIHSS score on admission, and medication use before admission

*p < 0.05,

**p < 0.01,

***p < 0.001

**Table 4:** In-hospital mortality rates for patients with intracerebral hemorrhage by eGFR levels on admission.

	eGFR mL/min/1.73 m^2^						
	Total N = 4036	≧90 N = 1149	60–89 N = 1746	30–59 N = 837	15–29 N = 135	<15 or dialysis N = 169	P for trend
Death, N (%)	201 (4.98)	35 (3.05)	74 (4.24)	53 (6.33)	19 (14.1)	20 (11.8)	<0.001
Withdrawal of life support, N (%)	84 (2.08)	11 (0.96)	24 (1.37)	26 (3.11)	4 (2.96)	19 (11.2)	<0.001

**Table 5:** Hazard ratios for mortality at 1 year after intracerebral hemorrhage.

	Crude		Adjusted^†^	
Variable	HR	(95% CI)	HR	(95% CI)
Sex (women vs. men)	1.10	(0.89, 1.36)	1.35	(1.07, 1.71)*
Age (year)	1.01	(1.01, 1.01)***	1.01	(1.002, 1.01)*
BMI (kg/m^2^)	0.94	(0.92, 0.96)***	0.96	(0.94, 0.98)
Smoking (past vs current)	0.99	(0.65, 1.49)	0.83	(0.54, 1.26)
Etiology				
Hypertension	1.00		1.00	
Non-hypertensive	1.53	(1.20, 1.94)***	1.73	(1.29, 2.31)***
Comorbidity				
Hypertension	0.74	(0.56, 0.96)*	0.79	(0.57, 1.11)
Atrial fibrillation	2.34	(1.46, 3.76)***	1.74	(1.05, 2.91)*
Previous stroke history	1.45	(1.15, 1.82)**	1.27	(0.99, 1.61)
Ischemic heart disease	1.70	(1.25, 2.30)***	1.49	(1.07, 2.06)*
Congestive heart failure	2.15	(1.11, 4.16)*	1.06	(0.52, 2.15)
Diabetes mellitus	1.36	(1.10, 1.68)**	1.11	(0.89, 1.40)
Systolic blood pressure, (mmHg)	1.01	(1.002, 1.01)***	1.01	(1.003, 1.009)***
Laboratory data				
Cholesterol (mg/dL)	1.00	(0.99, 1.00)	1.00	(1.00, 1.002)
Hb (g/dL)	0.85	(0.82, 0.89)***	0.96	(0.91, 1.01)
NIHSS score on admission	1.02	(1.01, 1.02)***	1.02	(1.02, 1.02)***
Medicine use before admission				
Antiplatelet drugs	0.82	(0.57, 1.19)	0.66	(0.44, 0.99)*
Warfarin	2.39	(1.49, 3.84)***	1.62	(0.97, 2.71)
Lipid lowering drugs	1.00	(0.64, 1.57)	0.71	(0.43, 1.17)

^†^Adjusted for age, sex, body mass index, hypertension, smoking, etiology of intracerebral hemorrhage, hypertension, atrial fibrillation, previous stroke history, ischemic heart disease, congestive heart failure, diabetes mellitus, systolic blood pressure, eGFR category, hemoglobin, cholesterol, NIHSS score on admission, and medicine use before admission

*p < 0.05,

**p < 0.01,

***p < 0.001

BMI, body mass index; Hb, hemoglobin; NIHSS, National Institutes of Stroke Scale

**Fig 3:**
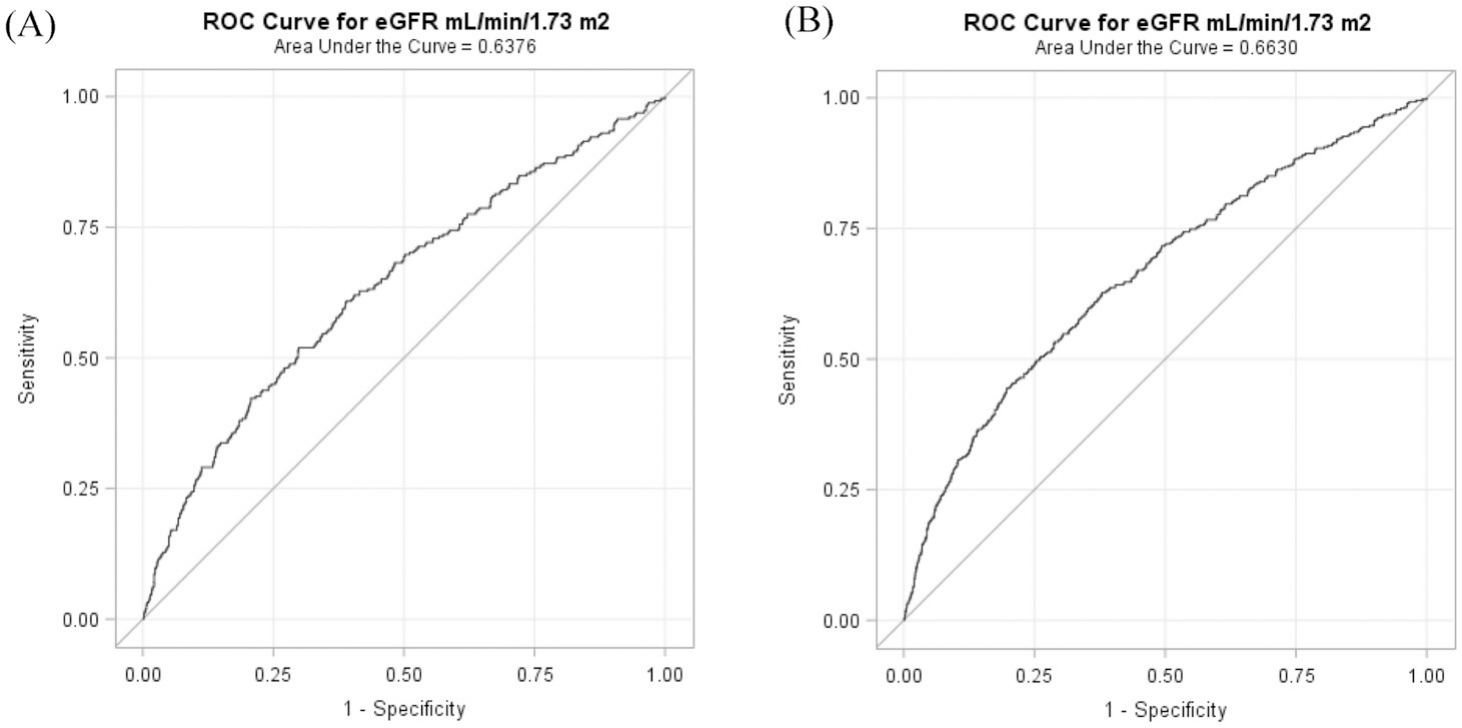
Area under the receiver operating characteristic curves for eGFR levels on admission in predicting 1-month (A) and 1-year (B) mortality in patients with intracerebral hemorrhage.

The areas under the ROC curves for the eGFR levels in predicting 1-month and 1-year mortality were 0.64 (Fig 3A) and 0.66 (Fig 3B) in patients with intracerebral hemorrhage, respectively.

## Discussion

Our study revealed that 394 of 4036 patients (9.76%) with intracerebral hemorrhage died within 1 year, and the majority of them [258 (65.5%)] died within 1 month. There was an independent graded association between eGFR levels and risks of both 1-month and 1-year mortalities after controlling for covariates of sex, age, stroke severity, and comorbidities. The risk of mortality increased as the eGFR declined.

Impaired renal function has been reported as an independent risk factor for mortality in patients with myocardial infarction, heart failure, or infectious diseases [23–26]. Renal dysfunction is also a known independent risk factor for mortality after stroke. A Scotland study followed 2042 patients with stroke for 7 years and found that reduced creatinine clearance (<51.27 mL/min) and elevated serum creatinine (≥1.35 mg/dL) could predict deaths in patients with acute stroke [8]. However, the study failed to demonstrate renal function as a significant predictor of mortality in hemorrhagic stroke because of the small size of fatal events [8]. A recent study using the TSR database also found that renal function could play a highly significant role in predicting mortality among patients with ischemic stroke [11]. An Israel study assessed risk factors associated with deaths for patients with acute stroke (ischemic or hemorrhagic) during 1-year follow-up [7]. Results demonstrated that eGFR was also a strong predictor of mortality and poor functional outcomes, such as nursing home dwelling and Barthel index ≦75 [7]. Hao et al. reported that an eGFR < 60 mL/min/1.73 m^2^ was a strong predictor of mortality and disability for hemorrhagic stroke but not for ischemic stroke [18]. Molshatzki et al. investigated 128 patients with an intracerebral hemorrhage and found that patients with moderate-to-severe reduction of eGFR (<45 mL/min/1.73 m^2^) had larger and lobar hematomas and hence higher one-year mortality rate (adjusted HR, 4.29; 95% CI 1.69–10.90) than those with eGFR > 60 mL/min/1.73 m^2^ [16]. A US study analyzing intracerebral hemorrhage data of 113,059 patients from the GET WITH The Guidelines-Stroke program showed that patients with declining eGFR had an increasing risk for in-hospital mortality [17]. The inpatient mortality was higher for those with eGFR < 15 mL/min/1.73 m^2^ (adjusted OR 2.22; 95% CI 2.04–2.43) relative to those with eGFR ≥ 90 mL/min/1.73 m^2^. On the contrary, a study analyzing the China National Stroke Registry found that low eGFR (<45 mL/min/1.73 m^2^) was independently associated with an increased risk of mortality in patients with diabetes complicated with ischemic events, but not in those with hemorrhagic stroke [19]. Our study focused on patients with intracerebral hemorrhage and found that eGFR levels could predict the risk of mortality in a graded relationship. These findings not only confirm the results of previous studies but also provide new information on the mortality prediction for intracerebral hemorrhage.

Renal function decline is associated with anemia, increased oxidative stress, abnormal apolipoprotein levels, inflammation, calcium-phosphate derangement, elevated uremic toxins, hypercoagulability, and impaired immunity [4, 27–29]. All these factors may contribute to the increased risks of adverse outcomes such as cardiovascular events, infection episodes, and deaths. These mechanisms may explain the graded association between impaired renal function and the risk of death in patients with hemorrhagic stroke.

Our data also indicated that most of the baseline comorbidities such as hypertension, ischemic heart disease, and diabetes were reversely associated with eGFR levels, indicating that patients with low eGFR levels were more critically ill. In addition, patients with a lower eGFR had higher NIHSS scores, indicating that impaired renal function could be associated with stroke severity.

The strength of this study is the use of a large sample size from stroke registry data with a representative group of patients with stroke in Taiwan. Thus, we could estimate the prognosis of real-world patients with intracerebral hemorrhage in Taiwan. In addition, we used the CKD-EPI equation to estimate the eGFR as it is better than the MDRD equation for Asian people [30]. The mortality rate after intracerebral hemorrhage in our study was lower than that in previous studies [16, 17, 31]. The reason is uncertain and probably related to different study populations. This study has several limitations. First, information on proteinuria was unavailable in the TSR database. We failed to evaluate eGFR and proteinuria simultaneously. Proteinuria is an important and independent risk factor for cardiovascular disease [5, 32]. Second, serum creatinine was measured and recorded once on admission, and it was confounded by acute illness. Therefore, it is difficult to determine the chronicity of renal dysfunction. Furthermore, some patients were excluded from the analysis because of missing information. The precision of measurement might be affected. However, the demographic characteristics, prevalence of comorbidities, laboratory values, and percentage of people using medication before admission were similar between the selected and excluded patients (S1 Table). Moreover, although data on hematoma volume and location of intracerebral hemorrhage were unavailable in this database, the NIHSS score to indicate stroke severity was used for adjustment in the multivariate analysis. The use of direct oral anticoagulants was also not recorded in this database. This is a limitation for external validity. Although information on the causes of deaths is unavailable, causes of 1-month mortality or in-hospital mortality could be mainly attributed to stroke and related complications. Thus, 1-month mortality or in-hospital mortality could be regarded as case fatality [31]. More than half of the patients who died within 1 year died at the hospitals (N = 201). The case fatality rates increased with the decline in eGFR levels. A recent study from Taiwan also revealed that stroke accounted for more than half of all deaths within 1 year in patients with hemorrhagic stroke [33].

In conclusion, there is an independent graded negative association between eGFR levels on admission and the death risk in patients with intracerebral hemorrhage. Patients with eGFR < 15 mL/min/1.73 m^2^ or on dialysis have a more than 4-fold increased hazard of death than those with eGFR ≥ 90 mL/min/1.73 m^2^. The eGFR level could be used as an early prognostic indicator for identifying high mortality risks for patients with intracerebral hemorrhage.

## Supporting information

S1 Table Baseline characteristics of excluded patients with intracerebral hemorrhage by eGFR levels on admission. (DOCX)

S2 Table List of Taiwan stroke registry investigators. (DOCX)
