# Supplementary material for: Renal function is associated with one-month and one-year mortality in patients with intracerebral hemorrhage
Source: PLoS One. 2023 Jan 26;18(1):e0269096. doi: 10.1371/journal.pone.0269096 (PMC9879419; doi:10.1371/journal.pone.0269096)
Supplement: S1 Table — (DOCX) [file pone.0269096.s001.docx]

| Table S1. Baseline characteristics of excluded patients with intracerebral hemorrhage by eGFR levels on admission | | | | | | | | | | | | | | | | | | | | | | | | | | |
| --- | --- | --- | --- | --- | --- | --- | --- | --- | --- | --- | --- | --- | --- | --- | --- | --- | --- | --- | --- | --- | --- | --- | --- | --- | --- | --- |
|  | eGFR mL/min/1.73 m^2^ | | | | | | | | | | | | | | | | | | | | | | | |  |  |
| Variable | Total  N = 10868 | | | ≧90  N = 3052 (28.1%) | | | | 60–89  N = 4451 (41.0%) | | | | 30–59  N = 2292 (21.1%) | | | | 15–29  N = 418 (3.85%) | | | | <15 or dialysis  N = 655 (6.03%) | | | | p-value | | |
| Men, N (%) | 7008 | (64.6) | 1989 | | (65.2) | | 2890 | | (64.9) | | 1463 | | (63.8) | | 271 | | (64.8) | | 395 | | (61.5) | | 0.4210 | | |  |
| Age, year, median (Q1, Q3) | 62.5 | (52.0–74.4) | | 53.5 | | (45.4–60.4) | | 67.1 | | (55.1–76.6) | | 71.9 | | (59.4–80.4) | | 66.9 | | (54.4–79.4) | | 62.1 | | (54.2–72.6) | | <0.001 | | |
| BMI, kg/m^2^, median (Q1, Q3) | 23.7 | (21.1–26.5) | | 23.9 | | (21.3–26.6) | | 23.7 | | (21.1–26.6) | | 23.7 | | (21.2–26.4) | | 23.0 | | (20.6–26.4) | | 22.5 | | (20.1–25.2) | | <0.001 | | |
| Smoking |  |  | |  | |  | |  | |  | |  | |  | |  | |  | |  | |  | | <0.001 | | |
| Current | 2265 | (20.8) | | 783 | | (25.7) | | 894 | | (20.1) | | 408 | | (17.8) | | 82 | | (19.6) | | 98 | | (15.0) | |  | | |
| Past | 942 | (8.67) | | 248 | | (8.13) | | 407 | | (9.14) | | 209 | | (9.12) | | 28 | | (6.70) | | 50 | | (7.63) | |  | | |
| Etiology, N (%) |  |  | |  | |  | |  | |  | |  | |  | |  | |  | |  | |  | | <0.001 | | |
| Hypertensive | 8649 | (79.6) | | 2223 | | (72.8) | | 3604 | | (81.0) | | 1914 | | (83.5) | | 343 | | (82.1) | | 565 | | (86.3) | |  | | |
| Non-hypertensive | 2219 | (20.4) | | 829 | | (27.2) | | 847 | | (19.0) | | 378 | | (16.5) | | 75 | | (17.9) | | 90 | | (13.7) | |  | | |
| Comorbidity, N (%) |  |  | |  | |  | |  | |  | |  | |  | |  | |  | |  | |  | |  | | |
| Hypertension | 8314 | (83.94) | | 2127 | | (75.83) | | 3450 | | (85.40) | | 1839 | | (88.54) | | 348 | | (90.39) | | 550 | | (91.97) | | <0.001 | | |
| AF | 267 | (2.46) | | 31 | | (1.02) | | 122 | | (2.74) | | 77 | | (0.71) | | 20 | | (4.78) | | 17 | | (2.60) | | <0.001 | | |
| Previous stroke history | 1911 | (17.58) | | 401 | | (13.14) | | 796 | | (17.88) | | 489 | | (21.34) | | 95 | | (22.73) | | 130 | | (19.85) | | <0.001 | | |
| Ischemic heart disease | 666 | (6.13) | | 75 | | (2.46) | | 256 | | (5.75) | | 209 | | (9.12) | | 44 | | (10.53) | | 82 | | (12.52) | | <0.001 | | |
| Congestive heart  failure | 140 | (1.29) | | 19 | | (0.62) | | 40 | | (0.90) | | 37 | | (1.61) | | 18 | | (4.31) | | 26 | | (3.97) | | <0.001 | | |
| Diabetes mellitus | 2540 | (23.37) | | 565 | | (18.51) | | 906 | | (20.35) | | 588 | | (25.65) | | 176 | | (42.11) | | 305 | | (46.56) | | <0.001 | | |
| Systolic blood pressure, mmHg | 177 | (152–205) | | 171 | | (146–1.98) | | 178 | | (154–204) | | 180 | | (153–209) | | 184 | | (149–211) | | 191 | | (160–222) | | <0.001 | | |
| Laboratory data, median (Q1, Q3) |  |  | |  | |  | |  | |  | |  | |  | |  | |  | |  | |  | |  | | |
| Cholesterol, mg/dL | 156 | (109–192) | | 162 | | (115–200) | | 154 | | (107–188) | | 157 | | (107–194) | | 145 | | (100–192) | | 138.5 | | (103–178.5) | | 0.009 | | |
| Hb, g/dL | 13.8 | (12.3–15.2) | | 14.2 | | (12.9–15.4) | | 14.2 | | (12.9–15.4) | | 13.6 | | (12.2–15.0) | | 11.8 | | (10.3–13.5) | | 10.5 | | (9.30–11.8) | | <0.001 | | |
| NIHSS score at admission, median (Q1, Q3) | 13 | (5–31) | | 12.0 | | (4.00–26.0) | | 13.0 | | (5.00–28.0) | | 15.0 | | (5.00–34.5) | | 21.0 | | (7.00–37.0) | | 19.5 | | (6.00–38.0) | | <0.001 | | |
| Medicine use prior to admission, N (%) |  |  | |  | |  | |  | |  | |  | |  | |  | |  | |  | |  | |  | | |
| Antiplatelet drugs | 832 | (7.66) | | 129 | | (4.23) | | 351 | | (7.89) | | 226 | | (9.86) | | 51 | | (12.20) | | 75 | | (11.45) | | <0.001 | | |
| Warfarin | 212 | (1.95) | | 39 | | (1.28) | | 82 | | (1.84) | | 67 | | (2.92) | | 16 | | (3.83) | | 8 | | (1.22) | | <0.001 | | |
| Lipid lowering drug | 293 | (2.70) | | 50 | | (1.64) | | 108 | | (2.43) | | 62 | | (2.71) | | 33 | | (7.89) | | 40 | | (6.11) | | <0.001 | | |
| eGFR, estimated glomerular filtration rate; Q1, 25th percentile; Q3, 75th percentile; BMI, body mass index; AF, atrial fibrillation; Hb, hemoglobin; NIHSS, National Institutes of Stroke Scale | | | | | | | | | | | | | | | | | | | | | | | | | | |
